# Complete Genome Sequences of Five Foot-and-Mouth Disease Viruses of Serotype A Isolated from Cattle in Nigeria between 2013 and 2015

**DOI:** 10.1128/genomeA.00039-18

**Published:** 2018-02-15

**Authors:** Frank Vandenbussche, Elisabeth Mathijs, Hussaini G. Ularamu, David O. Ehizibolo, Andy Haegeman, David Lefebvre, Annebel R. De Vleeschauwer, Steven Van Borm, Kris De Clercq

**Affiliations:** a Molecular Platform, Veterinary and Agrochemical Research Centre, Ukkel, Belgium; b FMD Laboratory, Viral Research Division, National Veterinary Research Institute (NVRI), Vom, Nigeria; c Vesicular and Exotic Diseases Unit, Veterinary and Agrochemical Research Centre, Ukkel, Belgium

## Abstract

The complete genome sequences of 5 foot-and-mouth disease viruses of serotype A are reported here. These viruses originate from outbreaks in northern Nigeria in 2013 to 2015 and belong to the A/AFRICA/G-IV lineage.

## GENOME ANNOUNCEMENT

Foot-and-mouth disease virus (FMDV) is a positive-sense single-stranded RNA virus of the genus *Aphthovirus* (family *Picornaviridae*) that causes a highly contagious vesicular disease in cloven-hooved animals. The virus is classified into 7 immunologically distinct serotypes (O, A, C, Asia 1, South African territories [SAT] 1, SAT 2, and SAT 3), each containing numerous genetically and geographically distinct evolutionary lineages or topotypes (1). FMDV is endemic in Nigeria, with 4 of the 7 serotypes (i.e., A, O, SAT 1, and SAT 2) reported during the last two decades (2–4). A recent study described the isolation and characterization of 9 FMDV-A strains (lineage A/AFRICA/G-IV) from northern Nigeria during 2013 to 2015 (5). In 2017, a closely related FMDV-A strain crossed the Sahara and caused outbreaks in Algeria (6, 7) and Tunisia (8). Here, we report the complete genome sequences of 5 FMDV-A strains that were isolated from epithelial tissue samples from Nigerian cattle showing typical foot-and-mouth disease (FMD) lesions.

All samples were collected from cattle herds located in northern Nigeria between 2013 and 2015. Total RNA was extracted from cell culture supernatant with a NucleoSpin RNA virus kit (Macherey-Nagel) and treated with Baseline-ZERO DNase (Epicentre) to remove host DNA. cDNA was synthesized according to the manufacturer’s instructions using SuperScript IV reverse transcriptase (Thermo Fisher Scientific), an anchored oligo(dT) primer, and an FMDV-specific internal primer. Sequencing libraries were prepared using a Nextera XT kit (Illumina) as described in the user’s manual. The fragment length distributions of the libraries were verified on a Bioanalyzer system (Agilent Technologies), and libraries were quantified using a library quantification kit (Kapa Biosystems). Sequencing was performed at the Biotechnology and Molecular Biology Platform (WIV-ISP, Brussels, Belgium) using a MiSeq reagent kit version 3 (Illumina) with 2 × 300-bp paired-end sequencing on a MiSeq system (Illumina).

Raw reads were trimmed using Trimmomatic version 0.36 with a quality cutoff of 15, a sliding window of 4 nucleotides, and a minimum length cutoff of 50 nucleotides (9). *De novo* assembly of the L fragment was performed using Iterative Virus Assembler (IVA) version 1.0.8 (10) and SPAdes (version 3.9.0) (11). The S fragment and the beginning and end regions of the L fragment were verified with Sanger sequencing. The genomes were annotated using the Genome Annotation Transfer Utility (GATU) (12) with the FMDV strain FMD/A/El-Fayoum/Egypt/2014 (GenBank accession number KP940474) serving as the reference genome.

Complete genome sequences were obtained for all 5 isolates, with lengths ranging from 8,126 to 8,135 nucleotides. The first 3 to 4 nucleotides of the genomes and the composition or length of the poly(C) tract could not be determined. The genomes were predicted to contain a single open reading frame (ORF) of 6,990 to 6,996 nucleotides encoding a polyprotein of 2,329 to 2,331 amino acids.

### Accession number(s).

The complete genome sequences for FMDV A/NIG/1/13, A/NIG/1/15, A/NIG/3/15, A/NIG/5/15, and A/NIG/6/15 have been deposited in GenBank under the accession numbers MG725872 to MG725876.
